# Harnessing selenocysteine to enhance microbial cell factories for hydrogen production

**DOI:** 10.3389/fctls.2022.1089176

**Published:** 2022-12-22

**Authors:** Armaan Patel, David W. Mulder, Dieter Söll, Natalie Krahn

**Affiliations:** 1 Department of Molecular Biophysics and Biochemistry, Yale University, New Haven, CT, United States; 2 National Renewable Energy Laboratory, Biosciences Center, Golden, CO, United States; 3 Department of Chemistry, Yale University, New Haven, CT, United States

**Keywords:** Selenocysteine, cell factories, biofuel, allo-tRNA, Selenoprotein, C1 microbes, hydrogenase, hydrogen production

## Abstract

Hydrogen is a clean, renewable energy source, that when combined with oxygen, produces heat and electricity with only water vapor as a biproduct. Furthermore, it has the highest energy content by weight of all known fuels. As a result, various strategies have engineered methods to produce hydrogen efficiently and in quantities that are of interest to the economy. To approach the notion of producing hydrogen from a biological perspective, we take our attention to hydrogenases which are naturally produced in microbes. These organisms have the machinery to produce hydrogen, which when cleverly engineered, could be useful in cell factories resulting in large production of hydrogen. Not all hydrogenases are efficient at hydrogen production, and those that are, tend to be oxygen sensitive. Therefore, we provide a new perspective on introducing selenocysteine, a highly reactive proteinogenic amino acid, as a strategy towards engineering hydrogenases with enhanced hydrogen production, or increased oxygen tolerance.

## 1 Introduction

C1-utilizing microbes, microorganisms which rely on one carbon molecule for survival, have been of interest to produce biofuels for industrial use (Du et al., 2011). Advances in metabolic engineering have led to the design of biosynthetic pathways as a means to efficiently use cellular machinery (Bar-Even et al., 2010). One application of these engineering strategies is to utilize the activity of [NiFe]- and [FeFe]-hydrogenases in C1 microbes. Hydrogenases are enzymes that catalyze the reversible oxidation of hydrogen and are used for hydrogen production, a renewable source of energy. To compete with existing chemical methods for hydrogen production, hydrogenases require a significant hydrogen production rate (Khanna and Lindblad, 2015). Furthermore, the highest hydrogen producing hydrogenases are also the most oxygen sensitive, reducing their efficiency within these microbial factories. Detailed studies on the factors driving hydrogen production and oxygen sensitivity have facilitated engineering strategies to overcome this (Wittkamp et al., 2018). More specifically, an investigation into the role of selenocysteine (Sec) in these key processes for the development of novel hydrogenases increases the applicability for industrial purposes (Marques et al., 2017; Evans et al., 2021).

Sec, a homolog of cysteine (Cys), is found in redox-associated enzymes across all domains of life (Li et al., 2014). With a single sulfur to selenium replacement compared to Cys, Sec retains similar chemistry but with enhanced chemical properties (i.e., increased nucleophilicity, decreased side-chain p*K*a, and increased oxidation which is often reversible) (Chung and Krahn, 2022). The distinct characteristics of this amino acid and its similarity to Cys suggests it is potential to affect the active site electronic properties, catalytic rate, or oxygen sensitivity of hydrogenases (Hondal et al., 2013; Marques et al., 2017; Evans et al., 2021).

**FIGURE 1:**
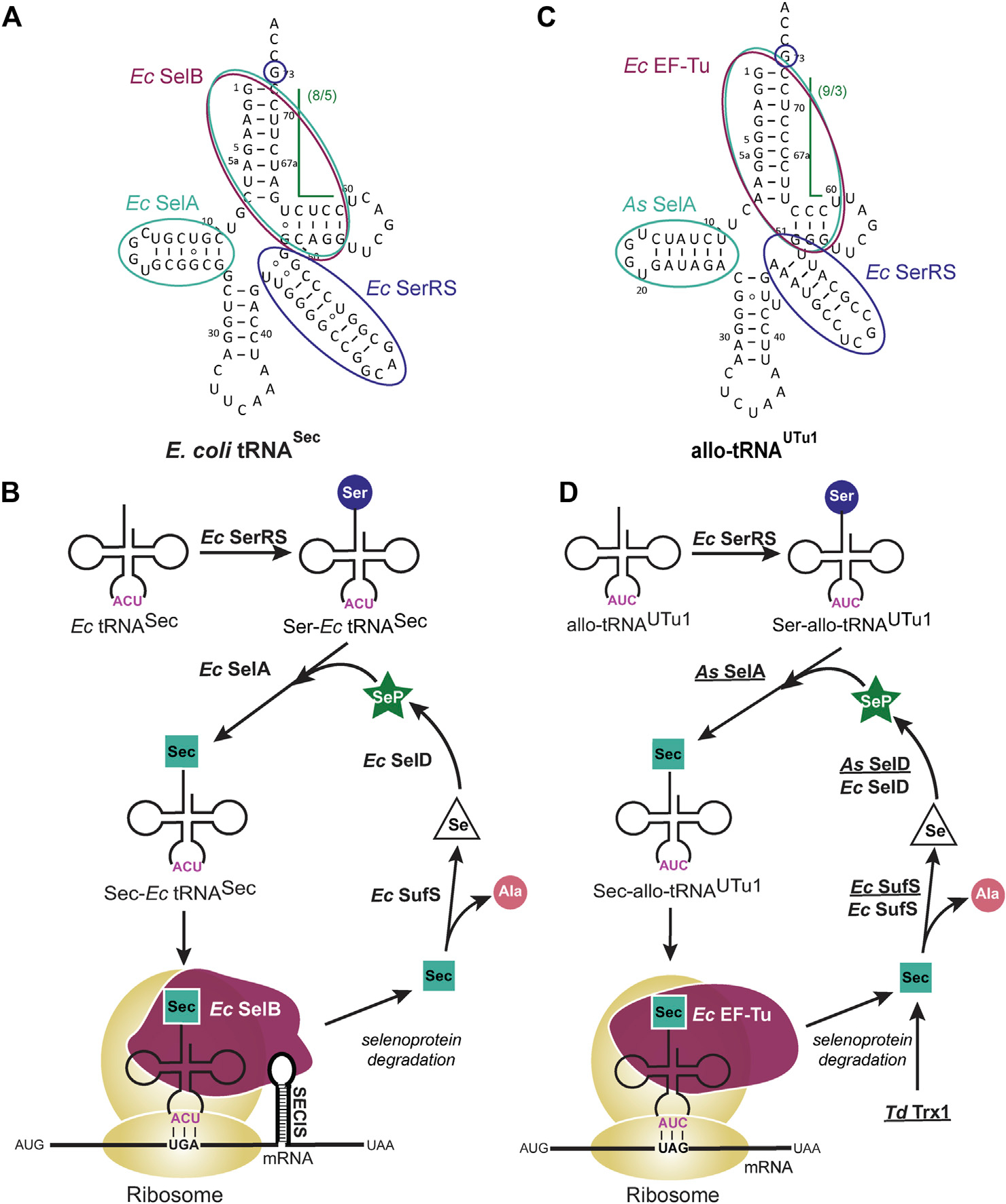
Selenocysteine (Sec) tRNA structure facilitates translation and insertion of Sec. **(A)** *Escherichia coli (Ec)* tRNA^Sec^ secondary structure highlights the features which facilitate its natural biosynthesis and insertion into the growing polypeptide chain. The long variable arm and G73 discriminator base (dark blue) are recognized by *Ec* seryl-tRNA synthetase (SerRS) for initial aminoacylation with Ser. Conversion of Serto Sec occurs through *Ec* selenocysteine synthase (SelA) which recognizes the D-arm and (8/5) acceptor domain (teal). This 13 bp acceptor domain discriminates tRNA^Sec^ from canonical tRNAs and enables recognition by a specialized elongation factor, *Ec* SelB (maroon), for insertion into the ribosome. **(B)** *Ec* SelB also recognizes an mRNA hairpin [Sec insertion sequence (SECIS) element] that is immediately downstream of the UGA codon for insertion of Sec. Degradation of used selenoproteins releases Sec which is a substrate for *Ec* Sec lyase (SufS), converting the amino acid to alanine (Ala) and releasing selenium (Se). Se is then converted to selenophosphate (SeP) by selenophosphate synthetase (SelD) where it re-enters the biosynthesis path. **(C)** allo-tRNA^UTu1^ secondary structure highlights the features which facilitate a simpler biosynthesis and insertion path for Sec in bacteria. The long variable arm and G73 discriminator base (dark blue) are recognized by *Ec* SerRS while the D-arm and (9/3) acceptor domain (teal) are recognized by *Aeromonas salmonicida* (As) SelA. Elongation of allo-tRNA^UTu1^ and insertion of Sec into the polypeptide chain occurs with *Ec* EF-Tu (maroon) which does not require the restrictive SECIS element. **(D)** The pSecUAG-Evol plasmid harbors additional enzymes that are recombinantly expressed (underlined) to promote Sec insertion in this simplified path. Selenoprotein degradation, including that of the additional *Treponema denticola* (Td) Trx1 releases Sec which is converted to Ala by both recombinant and endogenous SufS. To increase conversion of Se to SeP, both *As* SelD and *Ec* SelD are present.

Occurring naturally in bacteria, Sec is incorporated in proteins at a UGA codon that immediately precedes a hairpin loop (known as the Sec insertion sequence [SECIS] element) in the translated region of the mRNA. Biosynthesis of Sec occurs on tRNA^Sec^, where it is first aminoacylated with serine (Ser) by seryl-tRNA synthetase (SerRS) and then converted to Sec by selenocysteine synthase (SelA) (Fu et al., 2018). SelA uses selenophosphate as a substrate for this conversion, provided by selenophosphate synthetase (SelD) (Stock and Rother, 2009). The resulting Sec-tRNA^Sec^ is recognized by a specialized elongation factor (SelB) for peptide elongation in the ribosome. SelB resembles the canonical elongation factor EF-Tu, but with a C-terminal extension for interaction with the SECIS element (Stock and Rother, 2009). This complex and highly regulated path for insertion of Sec (Figures 1A, B) has been an obstacle for recombinant selenoprotein production (Fu et al., 2018).

In this perspective, we discuss the details of an emerging technology for site-specific Sec insertion in *Escherichia coli* and how it can be adapted to cell factories. We focus on applying these cell factories for hydrogen production, highlighting recent evidence on the novel properties imparted by Sec on hydrogenases.

## 2 Engineering tRNAs for SECIS-independent selenocysteine insertion

The complicated biosynthesis of Sec is facilitated by the tRNA^Sec^ structure, its main distinguishing factor being a 13 bp acceptor domain (acceptor stem and T-stem combined). This promotes recognition by SelB and reduces EF-Tu binding which favors the canonical 7/5 tRNA structure (12 bp acceptor domain) (Krahn et al., 2020). SelB also requires recognition of the SECIS element to facilitate insertion of Sec. This interaction controls the position of Sec within a protein, but it restricts the ability to overexpress these proteins or make novel selenoproteins (Cheng and Arnér, 2017). Therefore, to remove these restrictions, it was hypothesized that a tRNA could be engineered to biosynthesize Sec but be inserted in the ribosome by EF-Tu (Aldag et al., 2013; Miller et al., 2015; Thyer et al., 2015). Taking advantage of prior knowledge on the tRNA^Sec^ elements required to interact with each protein in this pathway (Krahn et al., 2020), one can strategically engineer a tRNA for SECIS-independent Sec insertion.

The discovery of allo-tRNAs, a group of tRNAs with novel secondary structure (Mukai et al., 2017), provided a scaffold for engineering a tRNA to insert Sec in an EF-Tu dependent manner. Allo-tRNAs are found to have 12 bp acceptor domains in a 9/3 or 8/4 configuration, unlike the 7/5 configuration of canonical tRNAs (Figure 1C). This alternate arrangement of the acceptor stem and T-stem does not appear to limit its binding to EF-Tu (Mukai et al., 2017), nor does it affect the distance between the acceptor stem and anticodon (Prabhakar et al., 2022). The anticodon is not a recognition element for any of the involved enzymes in Sec biosynthesis (Krahn et al., 2020), therefore it can be manipulated for readthrough of Sec at a UAG codon in addition to its natural suppression at a UGA codon (Mukai et al., 2017; Mukai et al., 2018). Moreover, some allo-tRNAs have features that resemble tRNA^Sec^, namely the G73 discriminator base, long variable arm, and unique D-arm. The first two features are key identity elements required for SerRS recognition, while the latter is recognized by SelA (Figure 1C) (Krahn et al., 2020). For these reasons, allo-tRNAs became the backbone for tRNA engineering in a rewired Sec translation path (Figure 1D) (Mukai et al., 2018).

Since it was confirmed that allo-tRNAs could be efficiently recognized by EF-Tu (Mukai et al., 2017), it was imperative to ensure that only Sec would get inserted into the protein. Unlike SelB which preferentially recognizes Sec-tRNA over Ser-tRNA (Leibundgut et al., 2005), EF-Tu only requires the presence of an amino acid on the tRNA (Schrader et al., 2011). Therefore, a closer investigation of the SelA and tRNA^Sec^ interaction was required to promote Ser to Sec conversion. In addition to the D-arm, *E. coli* SelA also recognizes the 13 bp acceptor domain of tRNA^Sec^ (Krahn et al., 2020). To promote interaction with allo-tRNAs, *Aeromonas salmonicida* (As), a close relative of *E. coli,* was found to have a SelA enzyme able to recognize 12 bp acceptor domain tRNAs. This major change accommodated Sec conversion on allo-tRNAs and was further enhanced by engineering the D-arm of allo-tRNAs to resemble that of *As* tRNA^Sec^ (Figure 1C) (Mukai et al., 2018).

The unique 9/3 structure of allo-tRNAs facilitate Sec biosynthesis and recognition by EF-Tu but does not provide any evidence as to how well it is accepted by the ribosome. Structural analysis and single-molecule translation studies revealed that the 9/3 acceptor domain does not interfere with translation. Instead, it was the rigidity of the variable arm affecting translocation of the allo-tRNA from the A- to the P-site of the ribosome. A single point mutation to disrupt the tertiary interaction in the central loop of the tRNA increased the flexibility of the variable arm to promote Sec insertion (Prabhakar et al., 2022).

The strategy of engineering allo-tRNAs for EF-Tu dependent translation has resulted in improved Sec incorporation compared to tRNAs engineered as a hybrid of tRNA^Sec^ and tRNA^Ser^ (Aldag et al., 2013; Miller et al., 2015; Thyer et al., 2015). The structure of allo-tRNAs has decoupled Sec translation from SelB and likewise the SECIS-element to facilitate Sec insertion into any position in the protein (Mukai et al., 2018). The versatility to site-specifically insert Sec and the enhanced chemical properties imposed by this amino acid makes it attractive for use in C1 microbial factories.

### 2.1 Adapting Sec translation in bacterial cell systems

The expression of allo-tRNAs in bacterial cell systems is mediated through a plasmid, specifically the pSecUAG-Evol plasmid series (Mukai et al., 2018; Chung et al., 2021). pSecUAG-Evol contains an allo-tRNA gene under an araC promoter plus other protein genes which have been found to enhance Sec insertion: i) *As* SelA to facilitate Ser to Sec conversion on allo-tRNAs and ii) *As* SelD, iii) *Treponema denticola (Td)* Trx1 and iv) mutant *E. coli* Sec lyase (SufS_C364A) to increase available selenium. With this plasmid, only two endogenous components from the host are required: i) SerRS for initial Ser aminoacylation and ii) EF-Tu to facilitate elongation in the ribosome. Endogenously expressed *E. coli* SelD and SufS are not essential to the path but can assist the recombinantly expressed proteins (*As* SelD and *Ec* SufS_C364A) to promote Sec insertion (Figure 1D).

The effectiveness of using allo-tRNAs for Sec insertion in *E. coli* suggests that they can also be adapted in bacterial cell factories. These factories can be composed of organisms from the genus *Clostridium* (Liew et al., 2016). For example, metabolically engineered *Clostridium autoethanogenum* is used to increase production of ethanol from carbon fixation (Liew et al., 2017). *C. autoethanogenum*, like *E. coli*, contains machinery for Sec incorporation, as seen by the presence of selenium containing formate dehydrogenase (Abubackar et al., 2015). Therefore, some of the proteins used for endogenous Sec insertion can also assist in recombinant expression. Furthermore, the >70% homology of both EF-Tu and SerRS in *C. autoethanogenum* compared to *E. coli* suggest that allo-tRNAs can potentially be serylated and recognized for elongation in these organisms.

The comparable machineries indicates that the pSecUAG-Evol plasmid system used in *E. coli* could be adapted for use in *C. autoethanogenum.* Optimization to determine the requirement or replacement of *As* SelD, *Td* Trx1 and *Ec* SufS_C364A to promote Sec insertion may be needed. These three proteins were added to increase the amount of available selenium in the cells without inducing cellular toxicity from additional selenium supplementation (Mukai et al., 2018). Codon usage in *E. coli* and *Clostridium* species also differs. Therefore, for optimal protein expression from the pSecUAG-Evol plasmid, codon optimization of these mRNA sequences would be preferable. As has been observed with *E. coli,* the expression levels of these additional proteins can be burdensome on the organism, therefore is should not be assumed that maximal expression levels are optimal (Mukai et al., 2018).

Another important consideration when adapting this system to bacterial cell factories, is the concentration and form of selenium donor that is being used. Some organisms tolerate different concentrations of sodium selenite (the selenium donor used in *E. coli*) before it inhibits their growth. However, other sources of selenium, such as selenomethionine can also be used and may be preferable for that organism (Mony and Larras-Regard, 2000).

## 3 Hydrogenases as a target for selenocysteine incorporation

**FIGURE 2:**
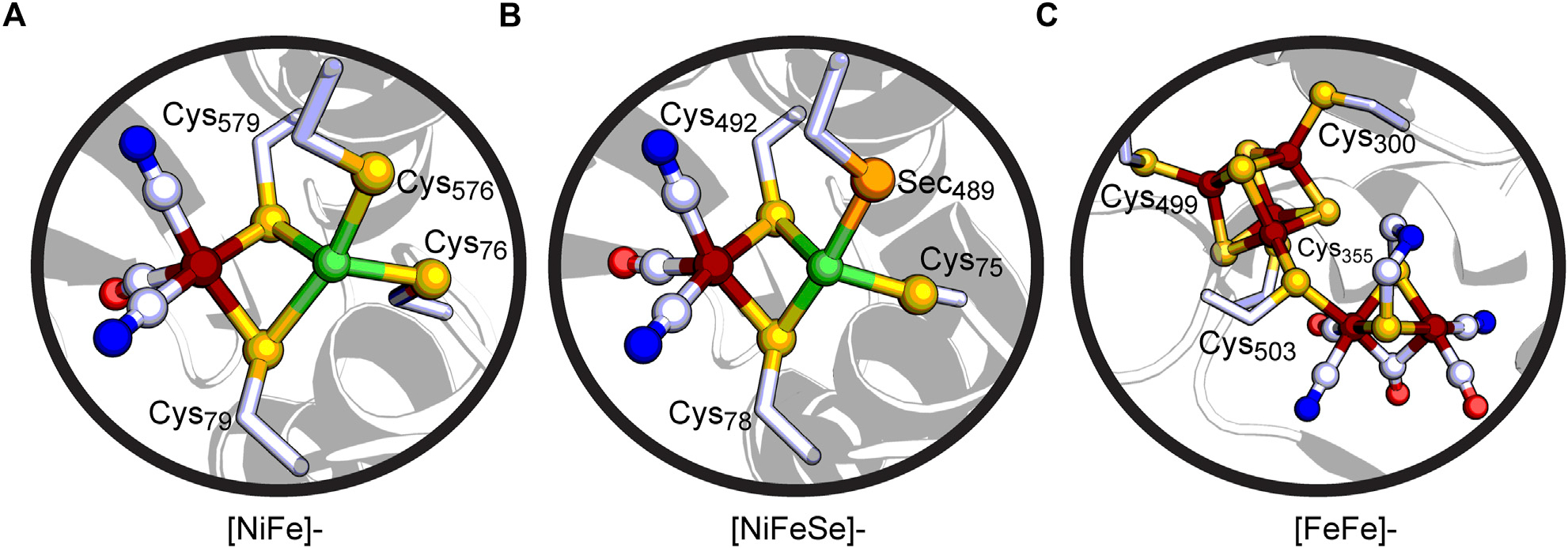
Cut-away views of **(A)** the active site of the [NiFe]-hydrogenase Hyd-1 from *Escherichia coli* (PDB: 5A4M), **(B)** the active site of the [NiFeSe]-hydrogenase from *Desulfovibrio vulgaris* Hildenborough (PDB: 5JSK), and **(C)** the H-cluster active site of the [FeFe]-hydrogenase CpI from *Clostridium pasteurianum* (PDB: 3C8Y). Coloring scheme: C, white; O, red; N, blue; S, yellow-orange; Se, orange; Ni, green; Fe, rust.

Hydrogenases, found across diverse microorganisms, are a class of enzymes that carry out essential functions in hydrogen metabolism (Vignais and Billoud, 2007; Calusinska et al., 2010; Peters et al., 2015; Greening et al., 2016). Two major classes of hydrogenases named according to the metal centers in their active site, [NiFe]- and [FeFe]-hydrogenases, catalyze the reversible oxidation of H_2_ gas from electrons and protons (2H^+^ + 2e^−^ ⇆ H_2_) (Figure 2) (Lubitz et al., 2014). A subset of [NiFe]-hydrogenases, the [NiFeSe]-hydrogenase, contains Sec in place of a Cys ligand in the active site (Garcin et al., 1999; Marques et al., 2010; Volbeda et al., 2013). A third class of hydrogenases, the Fe-hydrogenases, contain a mono-nuclear Fe active site and activates H_2_ in the presence of the substrate methylenetetrahydromethanopterin (Shima et al., 2008). Due to the ability of the [NiFe]- and [FeFe]-hydrogenases to achieve high reaction rates under ambient conditions, these enzymes and respective active-site structures serve as design models for bio-mimetic and bio-inspired catalysts (Simmons et al., 2014; Schilter et al., 2016; Dutta et al., 2018; Kleinhaus et al., 2021).

While the catalytic activation of H_2_ is reversible, the [NiFe]- and [FeFe]-hydrogenases often display a catalytic bias which is reflected by dis-proportionate reactions rates for the H_2_ oxidation and evolution directions (Mulder et al., 2021). This underscores the enzymes function in H_2_ metabolism, making them ideal targets for renewable H_2_ production and storage technologies through coupling to other reductive or oxidative reactions (Schuchmann et al., 2018). By example, electrons generated by the H_2_ oxidation reaction from [FeFe]-hydrogenase can be coupled to reductive processes such as CO_2_ reduction to formate (Schuchmann and Müller, 2013). Conversely, the H_2_ evolution reaction is often coupled to oxidation of mobile electron-carriers such as ferredoxin or NADPH in anaerobic fermentation. Green algae and cyanobacteria type hydrogenases play an important role in photosynthetic H_2_ production, through coupling electrons generated from the water splitting reaction to H_2_ evolution (Akhlaghi and Najafpour-Darzi, 2020; Kosourov et al., 2021).

### 3.1 [NiFe]- and [NiFeSe]-hydrogenases

[NiFe]-hydrogenases contain an active site with the Ni and Fe metals bridged together by Cys residues (Figure 2A) (Volbeda et al., 1995; Shafaat et al., 2013; Lubitz et al., 2014; Peters et al., 2015; Ogata et al., 2016). The Ni in the active site is coordinated by four Cys ligands: two bridging Cys thiolate ligands connect the Ni to the Fe and two terminal Cys thiolate ligands are located at the Ni. Furthermore, the Fe is coordinated by two CN^−^ molecules and one CO molecule. The [NiFeSe]-hydrogenases share a similar active site (Figure 2B), whereas one of the Cys ligands is Sec. Nearby non-coordinating conserved residues by the protein scaffold play important roles in tuning the active site for reversible H_2_ oxidation reaction through hydrogen-bonding interactions (Brooke et al., 2017). Conserved Asp and Arg residues found distal to the bridging Cys ligands are necessary for efficient H_2_ oxidation (Evans et al., 2016; Vansuch et al., 2020), whereas a conserved Glu residue adjacent to the terminal Cys acts as the universal proton gate to the active site (Dementin et al., 2004; Evans et al., 2018). Outside of the active site environment, there exist FeS clusters which allow for long-range transfer of electrons. The O_2_-tolerant [NiFe]-hydrogenases (Group 1D) contain a unique proximal [4Fe-3S] 6-Cys ligated cluster in relation to the active site that can reduce O_2_ to water, preventing a loss of catalytic activity (Fritsch et al., 2011; Goris et al., 2011; Shomura et al., 2011). On the other hand, O_2_-sensitive [NiFe]-hydrogenases do not have all 6 Cys residues present in this FeS cluster, which has been suggested to cause O_2_-sensitivity. [NiFeSe]-hydrogenases (Group 1A) are reported to have higher activity for producing H_2_ while [NiFe]-hydrogenases are typically biased for H_2_ oxidation (Parkin et al., 2008).

The [NiFe]-hydrogenases lose much of their catalytic activity in the presence of O_2_. In some [NiFe]-hydrogenases they regain activity once the O_2_ dissipates, but in others they require proton reduction before they become active again (Shafaat et al., 2013; Ogata et al., 2016). Those enzymes capable of oxidizing H_2_ in the presence of oxygen (albeit at a lower level) are deemed O_2_ tolerant. The catalytic mechanism of [NiFe]-hydrogenases has been well-studied for use in biotechnological applications, however the requirement to work in an aerobic environment reduces the feasibility of this endeavor.

As we begin to understand the role of Sec in [NiFeSe]-hydrogenases, there may be a solution unraveling to the O_2_ sensitivity roadblock. Initial studies looking at a natural [NiFeSe]-hydrogenase, investigated the effects of Sec to Cys substitution on its activity and O_2_ tolerance. The presence of a Cys instead of a Sec at the active site reduced Ni incorporation, H_2_ production activity, and O_2_ tolerance of the enzyme (Marques et al., 2017). In an opposite study, effects of a Cys to Sec substitution in a natural [NiFe]-hydrogenase were investigated (Evans et al., 2021). Regardless of the position of the Sec substitution in the active site, the H_2_ oxidation activity was reduced, and no H_2_ production activity was observed. On the other hand, substituting a Sec residue in the same position as what is observed in natural [NiFeSe]-hydrogenases, significantly increases the O_2_ tolerance (Evans et al., 2021).

These combined results suggest that the presence of Sec in the active site is not the only factor affecting a shift to H_2_ production activity. Rather the surrounding environment of the catalytic active site also influences the capability of [NiFe]-hydrogenases to oxidize or produce H_2_. However, the evidence is convincing that the presence of selenium increases O_2_ tolerance. This is the opposite to what one may expect given that selenium is readily oxidized. While this is true, it appears that the reversibility and ease in reduction of selenium oxides compared to sulfur oxides plays a role in this effect (Maroney and Hondal, 2018). One suggestion is that the selenium atom acts as a decoy, being preferentially oxidized, and therefore avoiding oxidation and inactivation of the [NiFe]-active site. Further investigation into the details of this chemical behavior still need to be uncovered. Conveying these two important details, [NiFeSe]-hydrogenases can be a solution towards the oxidation inactivation problem, and a promising target for growth in C1 microbes.

### 3.2 [FeFe]-hydrogenases

The second major group of hydrogenases, [FeFe]-hydrogenases, contain a complex metallocofactor active site termed the H-cluster and are known to be very active in H_2_ production (Lubitz et al., 2014; Peters et al., 2015). The H-cluster is comprised of a Cys ligated [4Fe-4S] cluster ([4Fe-4S]_H_) and binuclear iron site ([2Fe]_H_) (Figure 2C) (Peters et al., 1998; Nicolet et al., 1999). The two centers are bridged and electronically linked via a Cys thiolate ligand which is the only covalent attachment point for [2Fe]_H_ to the protein scaffold (Popescu and Münck, 1999). This differs from the structure of the [NiFe]-hydrogenase active site, which contains multiple bridging Cys ligands connecting the active site to the protein scaffold. Similar to the [NiFe]-hydrogenase active site, biologically unique CO and CN^−^ ligands coordinate the Fe atoms of [2Fe]_H_, in addition to a bridging azadithiolate ligand (Silakov et al., 2007; Berggren et al., 2013; Esselborn et al., 2013). The latter plays an important role in H_2_ activation. The bridgehead amine can function as a catalytic base (Fan and Hall, 2001) to the H^+^ binding site at the distal Fe atom of [2Fe]_H_, providing a key interaction point with a conserved proton-transfer pathway that terminates with a Cys within H-bonding distance of the ligand (Ginovska-Pangovska et al., 2014; Duan et al., 2018; Kisgeropoulos et al., 2022). Numerous site-directed mutagenesis studies have demonstrated the importance of the surrounding protein scaffold of the H-cluster in tuning its electronic properties and catalytic activity (Winkler et al., 2013; Stripp et al., 2022). Similar to [NiFe]-hydrogenases, long range potential effects on catalysis are possible through the presence of additional FeS clusters that function in electron-transfer (Greco et al., 2011; Rodriguez-Maciá et al., 2017; Caserta et al., 2018). These are present in different subgroups of [FeFe]-hydrogenases by means of ferredoxin-like binding domains present in a modular-like fashion (Mulder et al., 2011).

Attempts have been made to introduce exogenous hydrogenases into cyanobacteria that can either pair with the endogenous, bidirectional Hox [NiFe]-hydrogenase or operate in engineered strains devoid of Hox and other endogenous uptake [NiFe]-hydrogenases (Ducat et al., 2011; Gartner et al., 2012; Khanna and Lindblad, 2015; Avilan et al., 2018; Kosourov et al., 2021). Success was found by expressing an [FeFe]-hydrogenase from the energy demanding nitrogen-fixing *Clostridium acetobutylicum* in non-nitrogen-fixing *Synechococcus elongatus* sp. 7942 (Ducat et al., 2011). Without the need to use energy from the sun for nitrogen fixation, the sunlight to H_2_ conversion was increased. A significant challenge towards these types of approaches is the extreme O_2_ sensitivity of [FeFe]-hydrogenases. While certain [FeFe]-hydrogenases such as one from *Clostridium beijerinckii* (CbA5H) are emerging with unique O_2_ sensitivity properties (Morra et al., 2016; Corrigan et al., 2020; Winkler et al., 2021; Morra, 2022), the majority of characterized [FeFe]-hydrogenases are irreversibly inactivated by O_2_. As a result, biohydrogen production must be a delicate process to ensure that [FeFe]-hydrogenases are not inhibited by O_2_ produced from light-dependent reactions. This is typically approached by regular aerobic growth of cyanobacteria to generate internal stores of reductants before transferring to an anaerobic atmosphere (with inactivation of photosystem III), to facilitate hydrogenase activity (Ducat et al., 2011).

These efforts show the potential for increasing H_2_ production levels of [FeFe]-hydrogenases, though deeper understanding of the H_2_ metabolism in cyanobacteria is necessary (Khanna and Lindblad, 2015; Kosourov et al., 2021). A recurring problem is that cyanobacteria are unable to proceed with both light-dependent reactions and [FeFe]-hydrogenase activity at the same time. Taking into consideration the remarkable O_2_ tolerance gained by Sec substitution in the active site of a [NiFe]-hydrogenase, there is a prospective application for Sec insertion in the active site of [FeFe]-hydrogenases. Related studies have looked at selenium substitution at the sulfide positions of the H-cluster metal site (Noth et al., 2016; Kertess et al., 2017). This is made possible through semi-artificial reconstitution procedures which achieve an active [FeFe]-hydrogenase by addition of a synthetic [2Fe]_H_ cluster {[Fe_2_(SCH_2_NHCH_2_S)(CO)_4_(CN)_2_]^2−^} into an apo-form of the enzyme only containing [4Fe-4S]_H_ (Berggren et al., 2013; Esselborn et al., 2013). For one selenium derivative H-cluster containing selenium at the thiolate positions of the azadithiolate ligand (SeCH_2_NHCH_2_Se), a slight increase in catalytic bias toward H_2_ evolution was observed, however this was accompanied by a decrease in O_2_ stability compared to the native enzyme (Kertess et al., 2017). Another selenium derivative H-cluster containing selenium at the [4Fe-4S]_H_ sulfide positions ([4Fe-4Se]_H_) displayed similar catalytic rates for H_2_ evolution compared to the native enzyme (Noth et al., 2016).

## 4 Outlook

We have highlighted an emerging recombinant technology for site-specific Sec insertion into proteins using novel allo-tRNAs in a specially designed plasmid. Furthermore, we have shown its application in hydrogenases and emphasize the transferability of this system into microbial cell factories. The unique chemical properties of Sec makes its incorporation beneficial to engineering hydrogenases with increased or tuned catalytic activity and, perhaps more difficult, O_2_ tolerance.

Evidence thus far has investigated the incorporation of Sec in the active site, however other conserved structures such as FeS clusters that function in electron-transfer may also play critical roles in regulating tolerance to oxidative damage. One such example for certain types of [NiFe]-hydrogenases which confer extreme O_2_ tolerance, is the presence of a unique [4Fe-3S] cluster proximal to the active site that contains 6 conserved Cys residues (Dementin et al., 2004; Evans et al., 2018). O_2_ tolerance is lost upon two Cys to Gly mutations and growth of the organisms in high O_2_ concentrations was affected (Goris et al., 2011). Although the tendency has been to substitute Sec for Cys in the active site, this suggests that other FeS cluster electron-transfer centers which are widely present among diverse [NiFe]- and [FeFe]-hydrogenases may also be suitable targets.

While we stress the substitution of Sec in hydrogenases and use in H_2_ production, the unique chemical properties of Sec can be harnessed in any protein when engineering microbial cell factories. Specifically, enhancing catalytic properties to enzymes which use Cys in their active site is on the top of the list.

## Data Availability

The original contributions presented in the study are included in the article/Supplementary Material, further inquiries can be directed to the corresponding author.

## References

[R1] Abubackar HN, Veiga MC, and Kennes C (2015). Carbon monoxide fermentation to ethanol by *Clostridium autoethanogenum* in a bioreactor with no accumulation of acetic acid. Bioresour. Technol. 186, 122–127. doi:10.1016/j.biortech.2015.02.113 25812815

[R2] Akhlaghi N, and Najafpour-Darzi G (2020). A comprehensive review on biological hydrogen production. Int. J. Hydrogen Energy 45 (43), 22492–22512. doi:10.1016/j.ijhydene.2020.06.182

[R3] Aldag C, Bröcker MJ, Hohn MJ, Prat L, Hammond G, Plummer A, (2013). Rewiring translation for elongation factor Tu-dependent selenocysteine incorporation. Angew. Chem. Int. Ed. Engl. 52 (5), 1481–1485. doi:10.1002/ange.201207567 23193031 PMC3776052

[R4] Avilan L, Roumezi B, Risoul V, Bernard CS, Kpebe A, Belhadjhassine M (2018). Phototrophic hydrogen production from a clostridial [FeFe] hydrogenase expressed in the heterocysts of the cyanobacterium *Nostoc* PCC 7120. Appl Microbiol. Biotechnol 102 (13), 5775–5783. doi:10.1007/s00253-018-8989-2 29691627

[R5] Bar-Even A, Noor E, Lewis NE, and Milo R (2010). Design and analysis of synthetic carbon fixation pathways. Proc. Natl. Acad. Sci. U. S. A. 107 (19), 8889–8894. doi:10.1073/pnas.0907176107 20410460 PMC2889323

[R6] Berggren G, Adamska A, Lambertz C, Simmons TR, Esselborn J, Atta M, (2013). Biomimetic assembly and activation of [FeFe]-hydrogenases. Nature 499 (7456), 66–69. doi:10.1038/nature12239 23803769 PMC3793303

[R7] Brooke EJ, Evans RM, Islam ST, Roberts GM, Wehlin SA, Carr SB, (2017). Importance of the active site “canopy” residues in an O_2_-tolerant [NiFe]-hydrogenase. Biochemistry 56 (1), 132–142. doi:10.1021/acs.biochem.6b00868 28001048

[R8] Calusinska M, Happe T, Joris B, and Wilmotte A (2010). The surprising diversity of clostridial hydrogenases: A comparative genomic perspective. Microbiol. Read. 156 (6), 1575–1588. doi:10.1099/mic.0.032771-0 20395274

[R9] Caserta G, Papini C, Adamska-Venkatesh A, Pecqueur L, Sommer C, Reijerse E, (2018). Engineering an [FeFe]-hydrogenase: Do accessory clusters influence O_2_ resistance and catalytic bias? J. Am. Chem. Soc. 140 (16), 5516–5526. doi:10.1021/jacs.8b01689 29595965

[R10] Cheng Q, and Arnér ES (2017). Selenocysteine insertion at a predefined UAG codon in a release factor 1 (RF1)-depleted Escherichia coli host strain bypasses species barriers in recombinant selenoprotein translation. J. Biol. Chem. 292 (13), 5476–5487. doi:10.1074/jbc.m117.776310 28193838 PMC5392690

[R11] Chung CZ, and Krahn N (2022). The selenocysteine toolbox: A guide to studying the 21^st^ amino acid. Arch. Biochem. Biophys. 730, 109421. doi:10.1016/j.abb.2022.109421 36183842

[R12] Chung CZ, Miller C, Söll D, and Krahn N (2021). Introducing selenocysteine into recombinant proteins in Escherichia coli. Curr. Protoc. 1 (2), e54. doi:10.1002/cpz1.54 33566458 PMC7972002

[R13] Corrigan PS, Tirsch JL, and Silakov A (2020). Investigation of the unusual ability of the [FeFe] hydrogenase from *Clostridium beijerinckii* to access an O_2_-protected state. J. Am. Chem. Soc. 142 (28), 12409–12419. doi:10.1021/jacs.0c04964 32580545

[R14] Dementin S, Burlat B, De Lacey AL, Pardo A, Adryanczyk-Perrier G, Guigliarelli B, (2004). A glutamate is the essential proton transfer gate during the catalytic cycle of the [NiFe] hydrogenase. J. Biol. Chem. 279 (11), 10508–10513. doi:10.1074/jbc.m312716200 14688251

[R15] Du J, Shao Z, and Zhao H (2011). Engineering microbial factories for synthesis of value-added products. J. Ind. Microbiol. Biotechnol. 38 (8), 873–890. doi:10.1007/s10295-011-0970-3 21526386 PMC3142293

[R16] Duan J, Senger M, Esselborn J, Engelbrecht V, Wittkamp F, Apfel UP, (2018). Crystallographic and spectroscopic assignment of the proton transfer pathway in [FeFe]-hydrogenases. Nat. Commun. 9 (1), 4726. doi:10.1038/s41467-018-07140-x 30413719 PMC6226526

[R17] Ducat DC, Sachdeva G, and Silver PA (2011). Rewiring hydrogenase-dependent redox circuits in cyanobacteria. Proc. Natl. Acad. Sci. U. S. A. 108 (10), 3941–3946. doi:10.1073/pnas.1016026108 21368150 PMC3053959

[R18] Dutta A, Appel AM, and Shaw WJ (2018). Designing electrochemically reversible H_2_ oxidation and production catalysts. Nat. Rev. Chem. 2 (9), 244–252. doi:10.1038/s41570-018-0032-8

[R19] Esselborn J, Lambertz C, Adamska-Venkates A, Simmons T, Berggren G, Noth J, (2013). Spontaneous activation of [FeFe]-hydrogenases by an inorganic [2Fe] active site mimic. Nat. Chem. Biol. 9 (10), 607–609. doi:10.1038/nchembio.1311 23934246 PMC3795299

[R20] Evans RM, Ash PA, Beaton SE, Brooke EJ, Vincent KA, Carr SB, (2018). Mechanistic exploitation of a self-repairing, blocked proton transfer pathway in an O_2_-tolerant [NiFe]-hydrogenase. J. Am. Chem. Soc. 140 (32), 10208–10220. doi:10.1021/jacs.8b04798 30070475

[R21] Evans RM, Brooke EJ, Wehlin SA, Nomerotskaia E, Sargent F, Carr SB, (2016). Mechanism of hydrogen activation by [NiFe] hydrogenases. Nat. Chem. Biol. 12 (1), 46–50. doi:10.1038/nchembio.1976 26619250

[R22] Evans RM, Krahn N, Murphy BJ, Lee H, Armstrong FA, and Söll D (2021). Selective cysteine-to-selenocysteine changes in a [NiFe]-hydrogenase confirm a special position for catalysis and oxygen tolerance. Proc. Natl. Acad. Sci. U. S. A. 118 (13), e2100921118. doi:10.1073/pnas.2100921118 33753519 PMC8020662

[R23] Fan HJ, and Hall MB (2001). A capable bridging ligand for Fe-only hydrogenase: Density functional calculations of a low-energy route for heterolytic cleavage and formation of dihydrogen. J. Am. Chem. Soc. 123 (16), 3828–3829. doi:10.1021/ja004120i 11457119

[R24] Fritsch J, Scheerer P, Frielingsdorf S, Kroschinsky S, Friedrich B, Lenz O, (2011). The crystal structure of an oxygen-tolerant hydrogenase uncovers a novel iron-sulphur centre. Nature 479 (7372), 249–252. doi:10.1038/nature10505 22002606

[R25] Fu X, Söll D, and Sevostyanova A (2018). Challenges of site-specific selenocysteine incorporation into proteins by *Escherichia coli*. RNA Biol. 15 (4–5), 461–470. doi:10.1080/15476286.2018.1440876 29447106 PMC6103685

[R26] Garcin E, Vernede X, Hatchikian EC, Volbeda A, Frey M, and Fontecilla-Camps JC (1999). The crystal structure of a reduced [NiFeSe] hydrogenase provides an image of the activated catalytic center. Structure 7 (5), 557–566. doi:10.1016/s0969-2126(99)80072-0 10378275

[R27] Gartner K, Lechno-Yossef S, Cornish AJ, Wolk CP, and Hegg EL (2012). Expression of *Shewanella oneidensis* MR-1 [FeFe]-hydrogenase genes in *Anabaena* sp. strain PCC 7120. Appl. Environ. Microbiol. 78 (24), 8579–8586. doi:10.1128/aem.01959-12 23023750 PMC3502911

[R28] Ginovska-Pangovska B, Ho MH, Linehan JC, Cheng Y, Dupuis M, Raugei S, (2014). Molecular dynamics study of the proposed proton transport pathways in [FeFe]-hydrogenase. Biochim. Biophys. Acta 1837 (1), 131–138. doi:10.1016/j.bbabio.2013.08.004 23981729

[R29] Goris T, Wait AF, Saggu M, Fritsch J, Heidary N, Stein M, (2011). A unique iron-sulfur cluster is crucial for oxygen tolerance of a [NiFe]-hydrogenase. Nat. Chem. Biol. 7 (5), 310–318. doi:10.1038/nchembio.555 21390036

[R30] Greco C, Bruschi M, Fantucci P, Ryde U, and De Gioia L (2011). Mechanistic and physiological implications of the interplay among iron-sulfur clusters in [FeFe]-hydrogenases. A QM/MM perspective. J. Am. Chem. Soc. 133 (46), 18742–18749. doi:10.1021/ja205542k 21942468

[R31] Greening C, Biswas A, Carere CR, Jackson CJ, Taylor MC, Stott MB, (2016). Genomic and metagenomic surveys of hydrogenase distribution indicate H_2_ is a widely utilised energy source for microbial growth and survival. ISME J. 10 (3), 761–777. doi:10.1038/ismej.2015.153 26405831 PMC4817680

[R32] Hondal RJ, Marino SM, and Gladyshev VN (2013). Selenocysteine in thiol/disulfide-like exchange reactions. Antioxid. Redox Signal 18 (13), 1675–1689. doi:10.1089/ars.2012.5013 23121622 PMC3613276

[R33] Kertess L, Wittkamp F, Sommer C, Esselborn J, Rüdiger O, Reijerse EJ, (2017). Chalcogenide substitution in the [2Fe] cluster of [FeFe]-hydrogenases conserves high enzymatic activity. Dalton Trans. 46 (48), 16947–16958. doi:10.1039/c7dt03785f 29177350

[R34] Khanna N, and Lindblad P (2015). Cyanobacterial hydrogenases and hydrogen metabolism revisited: Recent progress and future prospects. Int. J. Mol. Sci. 16 (5), 10537–10561. doi:10.3390/ijms160510537 26006225 PMC4463661

[R35] Kisgeropoulos EC, Bharadwaj VS, Mulder DW, and King PW (2022). The contribution of proton-donor *pKa* on reactivity profiles of [FeFe]-hydrogenases. Front. Microbiol. 13, 903951. doi:10.3389/fmicb.2022.903951 36246213 PMC9563086

[R36] Kleinhaus JT, Wittkamp F, Yadav S, Siegmund D, and Apfel UP (2021). [FeFe]-Hydrogenases: Maturation and reactivity of enzymatic systems and overview of biomimetic models. Chem. Soc. Rev. 50 (3), 1668–1784. doi:10.1039/d0cs01089h 33305760

[R37] Kosourov S, Böhm M, Senger M, Berggren G, Stensjö K, Mamedov F, (2021). Photosynthetic hydrogen production: Novel protocols, promising engineering approaches and application of semi-synthetic hydrogenases. Physiol. Plant 173 (2), 555–567. doi:10.1111/ppl.13428 33860946

[R38] Krahn N, Fischer JT, and Söll D (2020). Naturally occurring tRNAs with non-canonical structures. Front. Microbiol. 11, 596914. doi:10.3389/fmicb.2020.596914 33193279 PMC7609411

[R39] Leibundgut M, Frick C, Thanbichler M, Böck A, and Ban N (2005). Selenocysteine tRNA-specific elongation factor SelB is a structural chimaera of elongation and initiation factors. EMBO J. 24 (1), 11–22. doi:10.1038/sj.emboj.7600505 15616587 PMC544917

[R40] Li F, Lutz PB, Pepelyayeva Y, Arnér ES, Bayse CA, and Rozovsky S (2014). Redox active motifs in selenoproteins. Proc. Natl. Acad. Sci. U.S.A. 111 (19), 6976–6981. doi:10.1073/pnas.1319022111 24769567 PMC4024873

[R41] Liew F, Martin ME, Tappel RC, Heijstra BD, Mihalcea C, and Köpke M (2016). Gas fermentation-a flexible platform for commercial scale production of low-carbon-fuels and chemicals from waste and renewable feedstocks. Front. Microbiol. 7, 694. doi:10.3389/fmicb.2016.00694 27242719 PMC4862988

[R42] Liew F, Henstra AM, Köpke M, Winzer K, Simpson SD, and Minton NP (2017). Metabolic engineering of Clostridium autoethanogenum for selective alcohol production. Metab. Eng. 40, 104–114. doi:10.1016/j.ymben.2017.01.007 28111249 PMC5367853

[R43] Lubitz W, Ogata H, Rüdiger O, and Reijerse E (2014). Hydrogenases. Chem. Rev. 114 (8), 4081–4148. doi:10.1021/cr4005814 24655035

[R44] Maroney MJ, and Hondal RJ (2018). Selenium versus sulfur: Reversibility of chemical reactions and resistance to permanent oxidation in proteins and nucleic acids. Free Radic. Biol. Med. 127, 228–237. doi:10.1016/j.freeradbiomed.2018.03.035 29588180 PMC6158117

[R45] Marques MC, Coelho R, De Lacey AL, Pereira IA, and Matias PM (2010). The three-dimensional structure of [NiFeSe] hydrogenase from *Desulfovibrio vulgaris* Hildenborough: A hydrogenase without a bridging ligand in the active site in its oxidised, ‘as-isolated” state. J. Mol. Biol. 396 (4), 893–907. doi:10.1016/j.jmb.2009.12.013 20026074

[R46] Marques MC, Tapia C, Gutiérrez-Sanz O, Ramos AR, Keller KL, Wall JD, (2017). The direct role of selenocysteine in [NiFeSe] hydrogenase maturation and catalysis. Nat. Chem. Biol. 13 (5), 544–550. doi:10.1038/nchembio.2335 28319099

[R47] Miller C, Bröcker MJ, Prat L, Ip K, Chirathivat N, Feiock A, (2015). A synthetic tRNA for EF-Tu mediated selenocysteine incorporation *in vivo* and *in vitro*. FEBS Lett. 589 (17), 2194–2199. doi:10.1016/j.febslet.2015.06.039 26160755 PMC4782793

[R48] Mony M-C, and Larras-Regard E (2000). Renal bioavailability of selenium after supplementation with different forms of selenium: Ion probe and mass spectrometry study. J. Trace Elem. Exp. Med. 13 (4), 367–380. doi:10.1002/1520-670x(2000)13:4<367::aid-jtra5>3.0.co;2-3

[R49] Morra S, Arizzi M, Valetti F, and Gilardi G (2016). Oxygen stability in the new [FeFe]-hydrogenase from *Clostridium beijerinckii* SM10 (CbA5H). Biochemistry 55 (42), 5897–5900. doi:10.1021/acs.biochem.6b00780 27749036

[R50] Morra S (2022). Fantastic [FeFe]-hydrogenases and where to find them. Front. Microbiol. 13, 853626. doi:10.3389/fmicb.2022.853626 35308355 PMC8924675

[R51] Mukai T, Vargas-Rodriguez O, Englert M, Tripp HJ, Ivanova NN, and Rubin EM (2017). Transfer RNAs with novel cloverleaf structures. Nucleic Acids Res. 45 (5), 2776–2785. doi:10.1093/nar/gkw898 28076288 PMC5389517

[R52] Mukai T, Sevostyanova A, Suzuki T, Fu X, and Söll D (2018). A facile method for producing selenocysteine-containing proteins. Angew. Chem. Int. Ed. Engl. 57 (24), 7215–7219. doi:10.1002/anie.201713215 29631320 PMC6035045

[R53] Mulder DW, Peters JW, and Raugei S (2021). Catalytic bias in oxidation-reduction catalysis. Chem. Commun. (Camb) 57 (6), 713–720. doi:10.1039/d0cc07062a 33367317 PMC9186002

[R54] Mulder DW, Shepard EM, Meuser JE, Joshi N, King PW, Posewitz MC, (2011). Insights into [FeFe]-hydrogenase structure, mechanism, and maturation. Structure 19 (8), 1038–1052. doi:10.1016/j.str.2011.06.008 21827941

[R55] Nicolet Y, Piras C, Legrand P, Hatchikian CE, and Fontecilla-Camps JC (1999). *Desulfovibrio desulfuricans* iron hydrogenase: The structure shows unusual coordination to an active site Fe binuclear center. Structure 7 (1), 13–23. doi:10.1016/s0969-2126(99)80005-7 10368269

[R56] Noth J, Esselborn J, Güldenhaupt J, Brünje A, Sawyer A, Apfel UP, (2016). [FeFe]-hydrogenase with chalcogenide substitutions at the H-Cluster maintains full H_2_ evolution activity. Angew. Chem. Int. Ed. Engl. 55 (29), 8536–8540. doi:10.1002/ange.201511896 27214763

[R57] Ogata H, Lubitz W, and Higuchi Y (2016). Structure and function of [NiFe] hydrogenases. J. Biochem. 160 (5), 251–258. doi:10.1093/jb/mvw048 27493211

[R58] Parkin A, Goldet G, Cavazza C, Fontecilla-Camps JC, and Armstrong FA (2008). The difference a Se makes? Oxygen-tolerant hydrogen production by the [NiFeSe]-hydrogenase from *Desulfomicrobium baculatum*. J. Am. Chem. Soc. 130 (40), 13410–13416. doi:10.1021/ja803657d 18781742

[R59] Peters JW, Lanzilotta WN, Lemon BJ, and Seefeldt LC (1998). X-ray crystal structure of the Fe-only hydrogenase (CpI) from *Clostridium pasteurianum* to 1.8 Angstrom resolution. Science 282 (5395), 1853–1858. doi:10.1126/science.282.5395.1853 9836629

[R60] Peters JW, Schut GJ, Boyd ES, Mulder DW, Shepard EM, Broderick JB, (2015). [FeFe]- and [NiFe]-hydrogenase diversity, mechanism, and maturation. Biochim. Biophys. Acta 1853 (6), 1350–1369. doi:10.1016/j.bbamcr.2014.11.021 25461840

[R61] Popescu CV, and Münck E (1999). Electronic structure of the H cluster in [Fe]-hydrogenases. J. Am. Chem. Soc. 121 (34), 7877–7884. doi:10.1021/ja991243y

[R62] Prabhakar A, Krahn N, Zhang J, Vargas-Rodriguez O, Krupkin M, and Fu Z (2022). Uncovering translation roadblocks during the development of a synthetic tRNA. Nucleic Acids Res. 50 (18), 10201–10211. doi:10.1093/nar/gkac576 35882385 PMC9561287

[R63] Rodriguez-Macia P, Pawlak K, Rüdiger O, Reijerse EJ, Lubitz W, and Birrell JA (2017). Intercluster redox coupling influences protonation at the H-clusterin [FeFe] hydrogenases. J.Am. Chem. Soc. 139 (42), 15122–15134. doi:10.1021/jacs.7b08193 28910086

[R64] Schilter D, Camara JM, Huynh MT, Hammes-Schiffer S, and Rauchfuss TB (2016). Hydrogenase enzymes and their synthetic models: The role of metal hydrides. Chem. Rev. 116 (15), 8693–8749. doi:10.1021/acs.chemrev.6b00180 27353631 PMC5026416

[R65] Schrader JM, Sakes ME, and Uhlenbeck OC (2011). “The specific interaction between aminoacyl-tRNAs and elongation factor Tu,” in Ribosomes. Editor Rodnina MV (Springer, Vienna: Wien and New York Spring-Verlag), 189–198. doi:10.1007/978-3-7091-0215-2_15

[R66] Schuchmann K, Chowdhury NP, and Müller V (2018). Complex multimeric [FeFe] hydrogenases: Biochemistry, physiology and new opportunities for the hydrogen economy. Front. Microbiol. 9, 2911. doi:10.3389/fmicb.2018.02911 30564206 PMC6288185

[R67] Schuchmann K, and Müller V (2013). Direct and reversible hydrogenation of CO_2_ to formate by a bacterial carbon dioxide reductase. Science 342 (6164), 1382–1385. doi:10.1126/science.1244758 24337298

[R68] Shafaat HS, Rüdiger O, Ogata H, and Lubitz W (2013). [NiFe] hydrogenases: A common active site for hydrogen metabolism under diverse conditions. Biochim. Biophys. Acta 1827 (8–9), 986–1002. doi:10.1016/j.bbabio.2013.01.015 23399489

[R69] Shima S, Pilak O, Vogt S, Schick M, Stagni MS, Meyer-Klaucke W, (2008). The crystal structure of [Fe]-hydrogenase reveals the geometry of the active site. Science 321 (5888), 572–575. doi:10.1126/science.1158978 18653896

[R70] Shomura Y, Yoon KS, Nishihara H, and Higuchi Y (2011). Structural basis for a [4Fe-3S] cluster in the oxygen-tolerant membrane-bound [NiFe]-hydrogenase. Nature 479 (7372), 253–256. doi:10.1038/nature10504 22002607

[R71] Silakov A, Reijerse EJ, Albracht SP, Hatchikian EC, and Lubitz W (2007). The electronic structure of the H-cluster in the [FeFe]-hydrogenase from *Desulfovibrio desufuricans:* A Q-band ^57^Fe-endor and HYSCORE study. J. Am. Chem. Soc. 129 (37), 11447–11458. doi:10.1021/ja072592s 17722921

[R72] Simmons TR, Berggren G, Bacchi M, Fontecave M, and Artero V (2014). Mimicking hydrogenases: From biomimetics to artificial enzymes. Coord. Chem. Rev. 270–271, 127–150. doi:10.1016/j.ccr.2013.12.018

[R73] Stock T, and Rother M (2009). Selenoproteins in archaea and gram-positive bacteria. Biochim. Biophys. Acta 1790 (11), 1520–1532. doi:10.1016/j.bbagen.2009.03.022 19344749

[R74] Stripp ST, Duffus BR, Fourmond V, Léger C, Leimkühler S, Hirota S, (2022). Second and outer coordination sphere effects in nitrogenase, hydrogenase, formate dehydrogenase, and CO dehydrogenase. Chem. Rev. 122 (14), 11900–11973. doi:10.1021/acs.chemrev.1c00914 35849738 PMC9549741

[R75] Thyer R, Robotham SA, Brodbelt JS, and Ellington AD (2015). Evolving tRNA^Sec^ for efficient canonical incorporation of selenocysteine. J. Am. Chem. Soc. 137 (1), 46–49. doi:10.1021/ja510695g 25521771 PMC4432777

[R76] Vansuch GE, Wu CH, Haja DK, Blair SA, Chica B, Johnson MK, (2020). Metal-ligand cooperativity in the soluble hydrogenase-1 from *Pyrococcus furiosus*. Chem. Sci. 11 (32), 8572–8581. doi:10.1039/d0sc00628a 34123117 PMC8163435

[R77] Vignais PM, and Billoud B (2007). Occurrence, classification, and biological function of hydrogenases: An overview. Chem. Rev. 107 (10), 4206–4272. doi:10.1021/cr050196r 17927159

[R78] Volbeda A, Amara P, Iannello M, De Lacey AL, Cavazza C, and Fontecilla-Camps JC (2013). Structural foundations for the O_2_ resistance of *Desulfomicrobium baculatum* [NiFeSe]-hydrogenase. Chem. Commun. (Camb) 49 (63), 7061–7063. doi:10.1039/c3cc43619e 23811828

[R79] Volbeda A, Charon MH, Piras C, Hatchikian EC, Frey M, and Fontecilla-Camps JC (1995). Crystal structure of the nickel-iron hydrogenase from *Desulfovibrio gigas*. Nature 373 (6515), 580–587. doi:10.1038/373580a0 7854413

[R80] Winkler M, Duan J, Rutz A, Felbek C, Scholtysek L, Lampret O, (2021). A safety cap protects hydrogenase from oxygen attack. Nat. Commun. 12 (1), 756. doi:10.1038/s41467-020-20861-2 33531463 PMC7854748

[R81] Winkler M, Esselborn J, and Happe T (2013). Molecular basis of [FeFe]-hydrogenase function: An insight into the complex interplay between protein and catalytic cofactor. Biochim. Biophys. Acta 1827 (8–9), 974–985. doi:10.1016/j.bbabio.2013.03.004 23507618

[R82] Wittkamp F, Senger M, Stripp ST, and Apfel UP (2018). [FeFe]-Hydrogenases: Recent developments and future perspectives. Chem. Commun. (Camb) 54 (47), 5934–5942. doi:10.1039/c8cc01275j 29726568

